# A Subtle Sister Mary Joseph Nodule in Metastatic Pancreatic Cancer

**DOI:** 10.3390/diagnostics16081190

**Published:** 2026-04-16

**Authors:** Mohammed Abdulrasak

**Affiliations:** 1 Department of Clinical Sciences, Lund University, 203 13 Malmo, Sweden; mohammed.abdulrasak@med.lu.se; 2 Department of Gastroenterology and Nutrition, Skåne University Hospital, 205 02 Malmo, Sweden

**Keywords:** metastatic disease, Sister Mary Joseph nodule, physical examination

## Abstract

A 47-year-old woman with metastatic pancreatic adenocarcinoma, diagnosed five months earlier and treated with palliative chemotherapy, was admitted with fever, jaundice, and right upper quadrant pain consistent with ascending cholangitis. Treatment with antibiotics was initiated and an endoscopic retrograde cholangiography was performed, whereby a biliary stent was placed to relieve malignant biliary obstruction. Physical examination revealed moderate ascites. Careful inspection of the umbilicus revealed a small nodular lesion located within the umbilical fold that became visible only after eversion of the umbilicus. The lesion had developed gradually over several weeks. Computed tomography confirmed the known pancreatic malignancy with metastatic disease and ascites. On re-review of the images, a small soft tissue nodule replacing the umbilicus was also visible. The lesion was clinically consistent with a Sister Mary Joseph nodule, an umbilical metastasis most commonly associated with advanced gastrointestinal or gynecologic malignancies. These lesions may arise through lymphatic or hematogenous spread or through direct extension into the umbilicus. This case highlights that umbilical metastases may be subtle and located within the umbilical fold, requiring careful physical examination to be detected.

**Figure 1:**
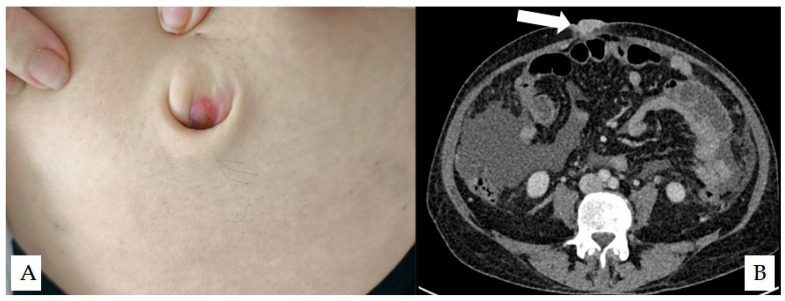
Clinical and radiological findings. (**A**) Umbilicus everted by the patient’s fingers demonstrating a small nodular lesion within the umbilical fold. (**B**) Axial contrast-enhanced computed tomography image showing metastatic pancreatic cancer with ascites. On re-review, a subtle soft tissue nodule (arrow) replacing the umbilicus can be identified. Sister Mary Joseph nodules represent cutaneous metastases to the umbilicus and are most commonly associated with advanced intra-abdominal malignancies, particularly of gastrointestinal or gynecologic origin [1]. Classically, these lesions present as firm, irregular, and often prominent umbilical masses that may distort or replace the normal umbilical contour [2]. In contrast, the lesion in the present case was small, located within the umbilical fold, and did not result in gross deformation of the umbilicus. Although a variety of benign conditions (such as dermatofibromas or keloid scars) may present with similar appearances, interpretation within the appropriate clinical context is essential [3]. The differential diagnoses for umbilical lesions are broad and may include umbilical hernia, omphalitis, and endometriosis alongside metastatic lesions [4]. In patients presenting with red-flag features such as unexplained weight loss, jaundice, or abdominal pain, the presence of an umbilical lesion should prompt consideration of underlying intra-abdominal malignancy [5]. Even subtle findings may therefore warrant further evaluation. Such presentations may be easily overlooked without careful physical examination, including eversion of the umbilicus. The novelty of this case lies not in the diagnosis itself, but in the subtle “intrafold” presentation, which required eversion of the umbilicus and re-review of imaging for detection. Although management was unchanged in this case, the finding supports the importance of careful physical examination as part of the clinician’s diagnostic armamentarium.

## Data Availability

Further information regarding the case is available on reasonable request from the corresponding author.
